# Estimating lime requirements for tropical soils: Model comparison and development

**DOI:** 10.1016/j.geoderma.2023.116421

**Published:** 2023-04

**Authors:** Fernando Aramburu Merlos, João Vasco Silva, Frédéric Baudron, Robert J. Hijmans

**Affiliations:** a Department of Environmental Science and Policy, University of California Davis, Davis, CA, United States; b Instituto de Innovación para la Producción Agropecuaria y el Desarrollo Sostenible (IPADS) Balcarce (INTA-CONICET), Balcarce, Buenos Aires, Argentina; c International Maize and Wheat Improvement Center (CIMMYT), Harare, Zimbabwe

**Keywords:** Acid soil, Exchangeable acidity, Aluminum saturation, Base saturation, pH, Calcium carbonate equivalent

## Abstract

Acid tropical soils may become more productive when treated with agricultural lime, but optimal lime rates have yet to be determined in many tropical regions. In these regions, lime rates can be estimated with lime requirement models based on widely available soil data. We reviewed seven of these models and introduced a new model (LiTAS). We evaluated the models’ ability to predict the amount of lime needed to reach a target change in soil chemical properties with data from four soil incubation studies covering 31 soil types. Two foundational models, one targeting acidity saturation and the other targeting base saturation, were more accurate than the five models that were derived from them, while the LiTAS model was the most accurate. The models were used to estimate lime requirements for 303 African soil samples. We found large differences in the estimated lime rates depending on the target soil chemical property of the model. Therefore, an important first step in formulating liming recommendations is to clearly identify the soil property of interest and the target value that needs to be reached. While the LiTAS model can be useful for strategic research, more information on acidity-related problems other than aluminum toxicity is needed to comprehensively assess the benefits of liming.

## Highlights

• Lime requirement models are useful to assess the potential benefits of liming.
• Lime rate estimates largely depend on the target soil property of the model.
• Stand-alone models that target soil pH have low accuracy.
• A new model was mathematically derived from the concept of acidity saturation.
• The new lime requirement model has better accuracy than all previous models.

## 1 Introduction

Acid soils may have a high concentration of phytotoxic elements such as aluminum and manganese in the soil solution and a low availability of phosphorus, calcium, and other plant nutrients (Kamprath, 1984). Soil acidity problems can be addressed with liming, the application of materials that react as a base and are rich in calcium and/or magnesium (Coleman et al., 1959). Liming has been practiced for centuries (Johnson, 2010), and its use is still expanding, particularly in tropical areas with acid soils. For example, it played a key role in the recent expansion of agriculture in the Brazilian Cerrado region on soils considered highly problematic for crop production (Goedert, 1983, Yamada, 2005).

The amount of lime required to adjust soil acidity depends on the soil, the target crop(s), and the liming materials used. In temperate regions, lime requirements are commonly estimated with locally-calibrated quick tests using buffer solutions (Goulding and de Varennes, 2016, Metzger et al., 2020, Rossel and McBratney, 2001, Sims, 1996). These tests can be developed by comparing the buffer’s response to the soil with the soil’s response to lime in field or incubation studies or by slow titrations. Both the soil testing and the lime application may be a relatively small expense in intensively-managed commercial farms, especially when lime is relatively cheap and because liming, when needed, increases the use efficiency of other inputs (de Wit, 1992). Moreover, large lime applications are often effective for several years due to the residual effects of the most-used liming materials and the buffering capacity of acid soils that reduces the risk of harm from applying more lime than is immediately required (Li et al., 2009).

This situation differs for many smallholder farmers in sub-Saharan Africa (Crawford et al., 2008) and other tropical regions (Sanchez and Salinas, 1981), where the soil testing, lime, and its application may be relatively expensive or inaccessible, and their benefit may be relatively small if fertilizer use is low. Moreover, empirical evidence on the effect of liming is often limited in these regions. Furthermore, methods that depend on measurements with buffer solutions must first be calibrated for each soil type and cannot be assumed to work without this required calibration. In these circumstances, models to estimate lime requirements from generally available soil property data (Hengl et al., 2017, Miller et al., 2021) could help better understand the potential benefits of liming. Lime requirement models could serve as a starting point to develop locally optimal liming recommendations. They could also provide strategic information on potential benefits and demand for lime for a region of interest.

Here, we provide a comprehensive review of lime requirement models for tropical acid soils that can be used with readily available soil data. The remainder of the paper is organized into seven sections. In Section 2, we introduce key concepts related to estimating lime requirements that have been a source of confusion and inconsistency. Section 3 presents the materials and methods used. In Section 4, we describe and discuss seven published lime requirement models for tropical soils and introduce a new model named LiTAS. The models are grouped by their target soil properties, and their accuracy is evaluated with experimental data from soil incubation studies. Section 5 presents a case study in which we run all models for a dataset of 303 African soil samples. We show substantial differences between models in the estimated lime requirement for acid tropical soils. In Section 6, we discuss the implications of our findings, and we conclude the paper in Section 7.

## 2 Key concepts and definitions

Soils are generally considered acid for crop production when they have a pH_(H2O)_ of 5.5 or less for most of the year (FAO, 2022, Sanchez, 2019). In the remainder of this paper, pH refers to the pH measured in a soil–water solution. This is the most commonly used method to measure pH and the pH measure available across the datasets we used (e.g., Teixeira et al., 2020a). Other methods to measure the soil pH include mixing the soil with an equivalent volume of 0.01 M CaCl_2_ or 1 M KCl. Soil pH_(CaCl2)_ is more stable against the seasonal changes in the electrolyte concentration of the soil solution that can affect pH_(H2O)_ measurements (Kissel et al., 2009). pH_(KCl)_ is used to measure the pH while accounting for the exchangeable acidity of the soil (Thomas, 1996). In acid soils, pH_(H2O)_ is generally higher than pH_(CaCl2)_ and pH_(KCl)_ (Kome et al., 2018, Sanchez, 2019).

Soils can be naturally acidic or become acidic because of agricultural practices such as the use of acidifying fertilizer and the removal of elements with harvested products. In the tropics, many soils in humid (and some subhumid) regions are inherently acid because intense weathering processes have resulted in the displacement and leaching of basic (i.e., non-acidic) exchangeable cations (Ca^2+^, Mg^2+^, K^+^, and Na^+^) and the accumulation of exchangeable acidity (Al^3+^ and H^+^). The main problem with soil acidity in the tropics is not the low pH as such, but rather the associated aluminum (Al) toxicity that constrains crop growth (Sanchez, 2019). The purpose of liming should therefore be to remove Al toxicity, considering the sensitivity of the target crops, together with alleviating other possible constraints such as Ca and Mg deficiencies (Kamprath, 1984, Sanchez, 2019), but not to increase pH for its own sake (Fageria and Baligar, 2008, Harter, 2007).

### 2.1 Target soil chemical properties

#### 2.1.1 Exchangeable acidity or aluminum

Acidity saturation is the fraction of the effective cation exchange capacity (*ECEC*) of the soil occupied by exchangeable acid cations (Al^3+^ and H^+^, extracted with a neutral unbuffered salt solution such as 1 M KCl). In tropical soils (except in histosols), nearly all exchangeable acidity is exchangeable Al^3+^; thus, Al saturation approximates acidity saturation (Deressa et al., 2020, Farina and Channon, 1991, Salinas, 1978). Therefore, acidity saturation is often used as a proxy for Al toxicity (Evans and Kamprath, 1970, Farina and Channon, 1991, Kamprath, 1980, Salinas, 1978, Smyth and Cravo, 1992). Many lime requirement models estimate the lime rate required to lower the acidity saturation to a target level that does not affect crop yield (Cochrane et al., 1980, Osmond et al., 2002, Yost et al., 1988).

The terms exchangeable acidity and exchangeable Al^3+^ have been used interchangeably in tropical soil literature, with the term exchangeable Al^3+^ more commonly used in older literature (Sanchez, 2019). Indeed, several authors of the lime requirement models reviewed here measured acidity saturation but referred to it as Al saturation (Cochrane et al., 1980, Kamprath, 1970). Consequently, some models were originally formulated for exchangeable Al^3+^ (and Al saturation) but derived from exchangeable acidity measurements.

#### 2.1.2 Exchangeable calcium and magnesium

Ca^2+^ and Mg^2+^ deficiencies coexist with Al toxicity problems in many acidic soils (Sanchez et al., 2019). Moreover, some highly weathered acid soils can have very low *ECEC* and, thus, low exchangeable Ca^2+^ and Mg^2+^ but low acidity saturation, resulting in Ca and Mg deficiencies without Al toxicity problems (Kamprath, 1984). Therefore, some lime requirement models based on acidity saturation also estimate the lime rate needed to cover these deficiencies (Sanchez, 2019, Teixeira et al., 2020b, van Raij, 1996). Organic or inorganic fertilizers applications can also be used to address such micronutrient deficiencies.

#### 2.1.3 Base saturation

A higher “base saturation” is an alternative to a lower acidity saturation in setting a target for alleviating soil acidity problems (Quaggio, 1983, van Raij, 1996). Base saturation *(V)* is the sum of all exchangeable bases (Ca^2+^, Mg^2+^, K^+^, and Na^+^) divided by the Cation Exchange Capacity at pH 7 (*CEC*_7_). *CEC*_7_ differs from *ECEC*, especially in acid soils, where *CEC*_7_ ≫ *ECEC*. For *ECEC*, exchangeable acid cations (Al^3+^ and H^+^) are extracted with a neutral unbuffered salt solution. In contrast, a pH 7 buffer solution is used for *CEC*_7_, which extracts both exchangeable and non-exchangeable acidity (for example, from hydroxy-Al organic matter complexes), comprising the potential acidity. The magnitude of the potential acidity of the soil depends on the type and amount of clay and organic matter. Although there is some inverse parallelism between acidity saturation and base saturation, these terms are not complementary because they have different denominators (*ECEC* and *CEC*_7_, respectively).

Contrary to Al toxicity and acidity saturation, there is no direct relation between base saturation and crop yields. Instead, a minimum base saturation threshold is defined such that, above it, no soil acidity problems are detected (Fageria and Baligar, 2008). Therefore, target base saturation levels must be defined locally for each crop type (van Raij, 1996).

#### 2.1.4 pH

Most lime requirement methods used in temperate regions target soil pH by estimating the lime rate required to raise the pH to a specific level (6 to 6.5 for most crops and soils) with locally-calibrated models (Goulding and de Varennes, 2016, Sims, 1996). In acid tropical soils, the yield of many crops may not be negatively affected by a soil pH as low as 5.0 depending on other soil chemical properties (Abruña et al., 1969, Bell, 1996, Pearson et al., 1977) and raising the pH can result in a loss of soil structure and other problems (Harter, 2007). Therefore, a target pH level is seldom used, and if it is used, it should be defined locally (Fageria and Baligar, 2008, Teixeira et al., 2020a).

Exchangeable acidity has a negative exponential association with soil pH (Supplementary Fig. 1). Very high exchangeable acidity values are only found in soils with a pH of 5.1 or lower, but not all soils with a low pH have high exchangeable acidity. Exchangeable acidity approaches 0 at a pH of 5.5, and there is virtually no exchangeable acidity above pH 6 (Supplementary Fig. 1) (Farina and Channon, 1991, Lollato et al., 2013, Sanchez, 2019). Therefore, a target pH of 5.5 should be high enough to address most Al toxicity problems.

#### 2.1.5 Phosphorus availability

Acid tropical soils usually have very low plant-available phosphorus because of the high P fixation capacity of Fe and Al oxides often found in these soils. Liming has the associated benefit of increasing P availability, which might result in significant yield responses, particularly when P fertilization is low (Salinas, 1978). However, liming can only temporarily relieve P deficiencies in soils with low P reserves (Smithson and Giller, 2002). Therefore, phosphorus availability is not considered a direct target of liming, and lime requirement models do not consider it. Yet, the increase in P availability can be an important reason for observing a yield increase in response to lime (Salinas, 1978).

### 2.2 Lime rate units

Lime rates (*LR*) are commonly expressed in charges per soil mass (e.g., meq per 100 g of soil or cmol_c_ per kg of soil, which are equivalent) or in the equivalent mass in tons (t, 1000 kg) of pure calcium carbonate (CaCO_3_) per unit area in hectares (ha). To transform lime rates between charges per soil mass and calcium carbonate mass per area, soil bulk density (*sbd*) and liming depth (*ld*) are needed. Lime rates in t ha^−1^ and cmol_c_ kg^−1^ are the same when *sbd* = 1 g cm^−3^ and *ld* = 20 cm. Thus, *LR* can be converted from charges per soil mass to calcium carbonate mass per area with Eq. (1), where *sbd* is expressed in g cm^−3^ and *ld* in cm.

(1) LRtCaCO3ha-1=LRcmolckgsoil-1×sbd×ld20

Many lime requirement models reviewed here provide lime rates in cmol_c_ kg^−1^. Therefore, when using these models to estimate lime rates in t ha^−1^, these must be transformed by considering the soil bulk density, lime incorporation depth, and the calcium carbonate equivalents (CCE) of the liming material to be applied. In addition, other models (Osmond et al., 2002, Yost et al., 1988) assume certain incorporation depth and soil bulk density and provide lime rates in t ha^−1^. However, these lime rates should be adjusted to account for potential differences between the assumed *ld* and *sbd* and the actual *ld* and *sbd*.

## 3 Materials and methods

A literature review was conducted to identify lime requirement models that only require soil properties available in soil databases to estimate lime rates for acid tropical soils. The terms “acid*” AND “soil*” AND (“lim* requirement” OR “lim* recommendation” OR “lim* rate”) were used in the Web of Science and Google Scholar databases to screen and retrieve relevant literature and references therein. Methods that required additional soil tests to measure the soil’s buffering capacity (e.g., Shoemaker et al., 1961) and methods developed for use in specific regions in temperate climates (e.g., Heckman et al., 2002, and Rossel and McBratney, 2001) were excluded. The search yielded seven models that can, in principle, be applied to a wide range of tropical soils. The identified models include five acidity saturation models, one base saturation model, and one pH model. These seven lime requirement models were reviewed and used to derive a new model based on acidity saturation. All models were implemented in an *R* package called “limer” (Aramburu Merlos, 2022) to facilitate their use and evaluation. The *R* package, data, and scripts used for analysis in this paper are available on GitHub (https://github.com/cropmodels/limer).

The lime requirement models were evaluated using data from four soil incubation studies that measured the effect of liming on exchangeable acidity and *ECEC* or acidity saturation (Ananthacumaraswamy and Baker, 1991, Cochrane et al., 1980, Kamprath, 1970, Teixeira et al., 2020a). Soil incubation studies are experiments in which soil samples are mixed with different amounts of lime and incubated under controlled conditions (∼ 30 °C and soil moisture at field capacity) for about a month to ensure that all lime reacts with the soil. The liming effect is assessed by measuring chemical soil properties before and after each lime treatment. Data from soil incubation studies that only measured the effect of liming on pH were not included because six out of the seven lime requirement models reviewed here need data on the effect of liming on exchangeable acidity and exchangeable bases to be evaluated.

The compiled data include strongly acidic to moderately acidic soils, four soil orders, and different tropical regions (Table 1). We included data for four Ultisols from the southeastern USA because they share features with the acidic Ultisols from humid tropical regions. The data from Kamprath, 1970, Cochrane et al., 1980, and Ananthacumaraswamy and Baker (1991) were readily available. However, only the lime rate estimates were available in Teixeira et al. (2020a), while the initial and final soil properties were not. We calculated the initial soil properties by back-solving the lime requirement formulas using the reported lime rates, which allowed us to recover the exact initial values. However, the final soil properties were estimated using the regression formulas provided in Teixeira et al. (2020a) supplementary information (R^2^ ≈ 0.9). Therefore, these values might not reflect all the variability in the original data.

**Table 1:** Description of the lime incubation studies data used to evaluate the performance of the lime requirement models. Data were extracted from Kamprath (1970) (Kamp), Cochrane et al. (1980) (Coch), Ananthacumaraswamy and Baker (1991) (Anan), and Teixeira et al. (2020a) (Teix). The range of values (minimum – maximum) is presented for lime rates (*LR*) and chemical soil properties. The number of *LR* treatments by soil type and the number of observations include the control treatments. *ECEC*: effective cation exchange capacity; AS (%): acidity saturation (exchangeable acidity divided by *ECEC*). *CEC*_7_: cation exchange capacity at pH 7. OM: organic matter. “-” indicates that this was not measured, while “m-” means it was measured but not available for each treatment (in which case we report the range of values reported in the original paper). Soil properties measured at the end of the experiments are in square brackets.

	Kamp	Coch	Anan	Teix
Year of study	1970	1980	1991	2020
# of soil types	4	2	3	22
*LR* treatments per soil type	5	5	4 or 5	8
# of observations	20	10	13	175
Soils region	North Carolina, USA	Colombia	Sri Lanka and Kenya	Minas Gerais, Brazil
Soil order	Ultisols	Ultisols, Oxisols	–	Inceptisols, Oxisols, Ultisols, Entisols
*LR* (cmol_c_ kg^−1^)	0.5 – 8.4	0.4 – 4	1 – 21.5	0.2 – 23.9
pH	4.5 – 4.7 [4.9 – 6]	–	–	4.1 – 5.3 [5.1 – 7.3]
AS (%)	53 – 82 [2 – 52]	68 – 86 [27 – 79]	49 – 81 [0 – 30]	9 – 96 [0 – 18]
*ECEC* (cmol_c_ kg^−1^)	1.1 – 7.8 [1.2 – 10.4]	3.4 – 4.4	6.3 – 9.1 [7 – 22.5]	0.5 – 3 [0.7 – 11.3]
*CEC*_7_ (cmol_c_ kg^−1^)	–	–	12 – 21	1.7 – 14
Clay content (%)	10 – 17	37 – 71	–	5 – 88 (m-)
OM (%)	2 – 7	–	–	0.4 – 8

We used all models to predict the lime rates required to reach the observed soil responses and compared these with the actual lime rates used in the experiments. For instance, the actual lime rate was compared with the predicted lime rate needed to reach the observed acidity saturation for models that use a target acidity saturation. The (dis)agreement between observed (*y*) and predicted (*ŷ*) lime rates was assessed with the root mean squared error ($\mathrm{RMSE}=\sqrt{\frac{1}{n}\sum_{1}^{n}{\left(y-\hat{y}\right)}^{2}}$), average model bias ($\mathrm{Bias}=\overset{¯}{y}-\overset{¯}{\hat{y}}$), and the concordance correlation coefficient (*r_c_*) (Lin, 1989). We repeated the accuracy assessment in three ways to account for the unbalanced number of observations between soil incubation studies and potential soil-incubation-study effects and to evaluate the models with data that was not used to calibrate them. First, we used all the available data to test each model, which might include the data used to calibrate the model. Second, we computed all accuracy metrics independently for each data set and reported their average so that all studies have the same weight on the evaluation. Third, we evaluated the models with data that was not used to calibrate them. When the data used to develop a particular model was the only data available for testing it, we used either a study-based or a six-fold cross-validation (James et al., 2013). For the study-based cross-validation, model coefficients were recalibrated with the data from three soil incubation studies and tested with the data from the remaining study, repeating the process for each dataset. When only data from one soil incubation study was available (as for the Teixeira model), we performed a six-fold cross-validation. We reported the average accuracy across folds or studies.

Lastly, we compared lime rate estimates from different models using soil data from the Africa Soil Profile Database (AfSP, Leenaars et al., 2014). The AfSP compiles georeferenced soil profile observations from many data sources. We selected soil samples with a pH between 3.5 and 6.5 that were tested for at least exchangeable acidity, *ECEC,* and *CEC_7_*, in which exchangeable acidity was extracted with 1 M KCl and *CEC_7_* measured in 1 M NH_4_OAc buffered at pH 7, ending with a total of 303 African soils. The origin of the selected soil samples is shown on a map in Supplementary Fig. 2, and their main properties are summarized in Supplementary Table 1. Lime requirements were estimated with the models described below for a lime incorporation depth of 20 cm.

## 4 Model description and evaluation

### 4.1 Acidity saturation models

This section describes five published lime requirement models based on acidity saturation (Kamprath, Cochrane, ACID4, NuMASS, and MG5) and introduces a new model (LiTAS), which were evaluated with data from four soil incubation studies (Ananthacumaraswamy and Baker, 1991, Cochrane et al., 1980, Kamprath, 1970, Teixeira et al., 2020a).

#### 4.1.1 Kamprath model

**Fig. 1:**
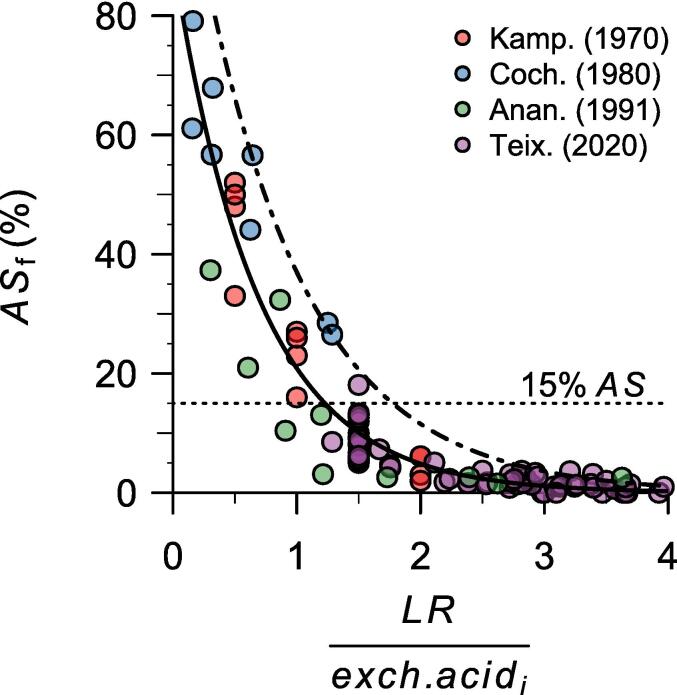
Acidity saturation after liming (AS_f_, %) as a function of the lime rate (*LR*, cmol_c_ kg^−1^) divided by the initial exchangeable acidity of the soil (*exch. acid*_*i*_, cmol_c_ kg^−1^) for soils with an initial acidity saturation >30%. Data were extracted from Kamprath (1970) (Kamp.), Cochrane et al. (1980) (Coch.), Ananthacumaraswamy and Baker (1991) (Anan.), and Teixeira et al. (2020a) (Teix.). The solid line is a negative exponential regression line $AS_{f}\left(\%\right)=95.7e^{-1.4LR/exch.a{\mathrm{cid}}_{i}}$ and the dot-dash line is a 95% negative exponential quantile regression line fitted with all the observations. Soil samples with *LR* > 4 × *exch. acid*_*i*_ had AS_f_ values ranging from 0 to 3.1%, with quartiles equal to 0, 0.2%, and 0.4% (these extreme values are not shown).

Kamprath (1970) measured the effect of different lime rates in a soil incubation study with four very acid soils (pH < 5, acidity saturation > 50 %). This study showed that acidity saturation does not decrease linearly with the amount of lime applied. When lime application rates are lower than the initial exchangeable acidity, acidity saturation is sharply reduced. However, for lime rates much greater than the initial exchangeable acidity, the fraction of lime charges that neutralizes exchangeable acidity is much lower because the lime also reacts with other forms of Al (e.g., organic-Al complex). Consequently, acidity saturation can be modeled as having a decreasing exponential response to lime that approaches zero at high lime rates (Fig. 1).

Kamprath (1970) concluded that a lime rate (cmol_c_ kg^−1^) of 1.5 times the initial exchangeable acidity (cmol_c_ kg^−1^) was enough to reduce the acidity saturation to at most 15%, which was considered to be a threshold below which most crops are not affected by acidity (Fig. 1). The suggested lime rate for sensitive crops needing an acidity saturation lower than 15%, such as beans (Abruña et al., 1969; Fageria et al., 2011; Kamprath, 1980), was twice the exchangeable acidity. Thus, Kamprath’s (1970) lime requirement model can be written as follows:

(2) $$LR\left(cmol_{c}kg_{\mathrm{soil}}^{-1}\right)=lf\times exch.a{\mathrm{cid}}_{i}\left(cmol_{c}kg_{\mathrm{soil}}^{-1}\right)$$

where *exch. acid_i_* is the initial exchangeable acidity of the soil, and *lf* is the lime factor, which is 1.5 for most staple crops (e.g., cereals) and 2 for beans and other crops sensitive to acidity, including many vegetable and fruit crops (Alvarez and Ribeiro, 1999).

This simple model worked well for almost all the experimental data available from the four studies (Fig. 1). Out of 21 very acid soils (acidity saturation, *AS*, between 30% and 97%) that received a lime rate of exactly 1.5 times the initial exchangeable acidity, only one ended with an acidity saturation >15%, but it was very close to that value (18%). Furthermore, all soil samples with a lime rate of at least twice the initial exchangeable acidity had a final acidity saturation of 6% or less. Hence, when the goal of liming is to reduce the acidity saturation to a level that does not affect crop growth, liming is only needed when the acidity saturation is above 15% (or 5% for sensitive crops). In such cases, lime rates of 1.5 (or two for sensitive crops) times the initial exchangeable acidity would suffice for most tropical soils.

Modifications of the Kamprath (1970) model were used in different regions of Brazil (Lopes et al., 1991) and Ethiopia (Alemu et al., 2022). For instance, in Minas Gerais, Brazil, a *lf* of 2 was recommended for most soil types, except for sandy soils (*lf* = 1) and clay soils (*lf* = 3; Lopes et al., 1991). These adjustments may account for differences in soil bulk density, as the modified formulas gave lime requirements in tons per hectare. Furthermore, all these modified models added a second term to account for possible Ca and Mg deficiencies, as was done in the Minas Gerais 5 model (MG5, Section 4.1.5).

#### 4.1.2 Cochrane model

Cochrane et al. (1980) introduced the concept of *target acidity saturation (TAS)* to estimate lime rates (originally called *required percentage Al saturation*, see Section 2.1.1). Considering the great variability in acidity saturation tolerance among and within crops (Kamprath, 1980, Lollato et al., 2019), Cochrane et al. (1980) developed a model to estimate the lime rate needed to reduce the acidity saturation to a specific target for a particular crop.

To derive their formula, Cochrane et al. (1980) started with a hypothetical situation where all lime reacts with the exchangeable acidity; thus, the *ECEC* itself does not change as the decrease in exchangeable acidity was assumed to equal the increase in exchangeable bases. In this scenario, the required lime rate to reach a given acidity saturation would be $\mathrm{LR}=exch.a{\mathrm{cid}}_{i}-exch.a{\mathrm{cid}}_{f}=exch.a{\mathrm{cid}}_{i}-\left(TAS/100\right)\times ECEC$. The target acidity saturation (*TAS*, %) is divided by 100 to change it to a fraction, and the subscript *i* indicates the initial and *f* the final values. The unit of *LR*, *exch. acid,* and *ECEC* is cmol_c_ kg^−1^.

The original formula uses the sum of exchangeable acidity (H^+^ and Al^3+^), Ca^2+^, and Mg^2+^ instead of *ECEC* because these were the cations measured by Kamprath (1970). The concentration of other bases, such as K^+^ and Na^+^, was considered negligible, as these are normally very low in acid soils. Thus, the sum of exchangeable acidity, Ca^2+^, and Mg^2+^ was considered equivalent to the *ECEC*. We present the formula using *ECEC*, noting that *ECEC* might not always include all cations but should always include the exchangeable Al^3+^, Ca^2+^, and Mg^2+^, as these are the most abundant cations in acid soils. If data on exchangeable K^+^ and Na^+^ are available, they might be included depending on which exchangeable cations were considered for the *TAS*.

Since not all the applied lime reacts with the exchangeable acidity, the formula is multiplied by a lime factor (*lf*) that equals 1.5 or 2 depending on the relation between initial exchangeable acidity, *TAS*, and *ECEC*. The authors defined the following rule: “*factor 1.5 is replaced by 2 when the estimated liming requirement using the factor 1.5 is greater than the chemical lime equivalent of the exchangeable Al* (acidity)*.”* Thus:

$$LR\left(cmol_{c}kg_{\mathrm{soil}}^{-1}\right)=lf\times \left[exch.a{\mathrm{cid}}_{i}-\left(TAS/100\right)\times ECEC_{i}\right]$$

(3) lf=1.5,if1.5×exch.acidi-TAS100×ECECi≤exch.acidi2,if1.5×exch.acidi-TAS100×ECECi>exch.acidi

Which can be simplified as lf=1.5,ifTAS≥ASi32,ifTAS<ASi3

Where *AS_i_* is the initial acidity saturation. In other words, when the target acidity saturation is less than one-third of the initial saturation, the lime factor is 2; otherwise, it is 1.5. For example, for soils with an initial acidity saturation of 60%, *lf* = 1.5 when *TAS* ≥ 20% and *lf* = 2 when *TAS* < 20%.

**Fig. 2:**
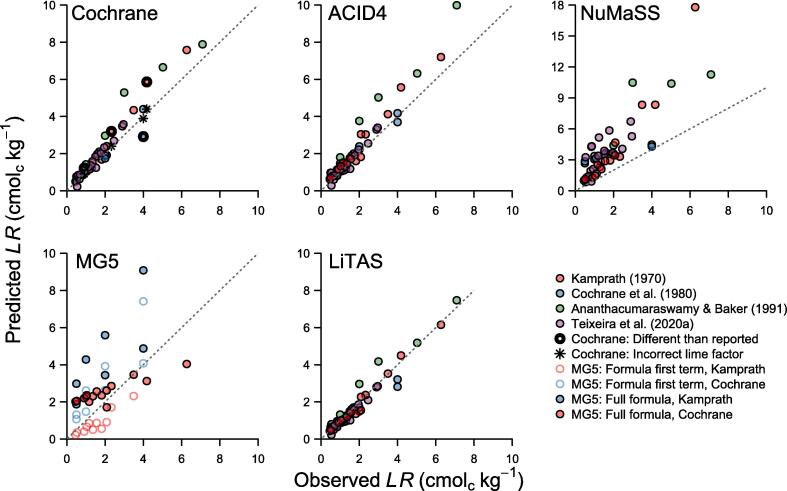
Observed and predicted lime rates (*LR*, cmol_c_ kg^−1^) to reach the exchangeable acidity saturation obtained with the observed lime rates for five lime requirement estimation models based on a target acidity saturation (Cochrane, ACID4, NuMaSS, MG5 and LiTAS). Observed data were extracted from Kamprath, 1970, Cochrane et al., 1980, Ananthacumaraswamy and Baker, 1991, and Teixeira et al. (2020a). Samples with a final acidity saturation of < 5% were excluded. In the Cochrane et al. (1980) model, thick points are values computed with Eq. (3) that are different from the values reported in Cochrane et al. (1980), and asterisks are values reported by Cochrane et al. (1980) that did not follow their model (incorrect lime factor). In the Minas Gerais 5th approximation model (MG5), filled circles were predicted using the complete formula, and empty circles by only considering its first term (acidity saturation requirements). Teixeira et al., 2020a, Ananthacumaraswamy and Baker, 1991 did not report soil texture; therefore, these data were not used with MG5. The gray dashed line is the identity function (Predicted *LR* = Observed *LR*).

Notably, when *TAS* = 0%, the required lime rate according to the Cochrane et al. (1980) model is twice the initial exchangeable acidy, just like the Kamprath (1970) model for sensitive crops. For that reason, Cochrane et al. (1980) suggested that their formula should not be evaluated for lime rates greater than twice the initial exchangeable acidity. Such lime rates result in about 5% acidity saturation or less (Fig. 1). Therefore, we recommend restricting the use of the Cochrane et al. (1980) model (and any other acidity saturation model) to a *TAS* ≥ 5%. Accordingly, we only evaluated models based on *TAS* for cases in which liming led to a final *AS* ≥ 5%, as lower *AS* values should not be the target of these models (Fig. 2). A model with a target pH of 6 might be more appropriate for extremely sensitive crops requiring an acidity saturation of < 5%, as exchangeable acidity is negligible at this pH (Supplementary Fig. 1).

We found several instances in the literature where the rule of changing the lime factor at low *TAS* in Eq. (3) was misused or ignored. First, Cochrane et al. (1980) inconsistently applied this rule when testing the performance of their model, perhaps to improve its apparent accuracy (Fig. 2). Second, later modifications and references to this model did not include the rule (Alvarez and Ribeiro, 1999, Osmond et al., 2002, Yost et al., 1988). For instance, Sanchez, 2019, Fageria and Baligar, 2008 described the formula with a unique *lf* = 1.8, which results from multiplying the original *lf* of 1.5 by 1.2 to express the *LR* in tons per hectare by assuming a soil bulk density (*sbd*) of 1.2 g cm^−3^ and a lime incorporation depth (*ld*) of 20 cm (Eq. (1)). Despite these inconsistencies, the Cochrane et al. (1980) model has high accuracy, even when evaluated with independent data (Table 2), and it represented a breakthrough in computing lime requirements. All subsequent models based on *TAS* derive from it.

**Table 2:** Accuracy metrics of lime requirement models. The models were evaluated in their capacity to predict the lime rate used to reach a given soil response. Accuracy metrics were computed with all data and with independent data (i.e., data not used to calibrate the model). Study-based cross-validation was used to evaluate the new model (LiTAS) and six-fold cross-validation for the Teixeira et al. (2020b) model. The accuracy metrics were the concordance correlation coefficient (*r*_c_), the average model bias (observed minus predicted), the root mean square error (RMSE), and the average RSME across datasets (RMSE_avg_). The closer the *r*_c_ is to 1, the better, and the closer the Bias, RMSE, and RMSE_avg_ are to 0, the better. The accuracy metrics of the Quaggio model are also reported for a final base saturation equal to or below 50%.

Target	Model	All data				Independent data			
		*r*_c_	Bias	RMSE	RMSE_avg_	*r*_c_	Bias	RMSE	RMSE_avg_
Acidity saturation	LiTAS	0.97	0.07	0.36	0.42	0.96	0.09	0.41	0.48
	Cochrane	0.93	−0.30	0.61	0.70	0.92	−0.36	0.63	0.84
	ACID4	0.91	−0.37	0.70	0.77	0.91	−0.37	0.70	0.77
	NuMaSS	0.46	−2.19	2.94	3.09	0.46	−2.19	2.94	3.09
	MG5	0.41	−1.17	1.91	1.96	0.41	−1.17	1.91	1.96
Base saturation	Quaggio	0.78	0.99	2.25	1.66	0.78	0.99	2.25	1.66
	Quaggio (50%)	0.90	0.17	0.63	0.75	0.90	0.17	0.63	0.75
pH	Teixeira	0.65	0.60	1.33	1.33	0.47	0.21	1.38	1.38

#### 4.1.3 ACID4 model

Yost et al. (1988) developed the ACID4 expert system to estimate lime requirement in the humid tropics. They used the Cochrane et al. (1980) formula with a fixed lime factor (*lf*) and a unit conversion from cmol_c_ kg^−1^ to t ha^−1^. Based on preliminary data from Sitiung, Indonesia, Yost et al. (1988) estimated that 0.53 cmol_c_ of exchangeable acidity was neutralized per cmol_c_ of CaCO_3_ and computed the *lf* as the inverse of that fraction (1/0.53 = 1.9). The accuracy of their model was slightly lower than that of the Cochrane et al. (1980) model (Fig. 2 and Table 2).

To convert the results from cmol_c_ kg^−1^ to tons of CaCO_3_ per ha, Yost et al. (1988) changed the *lf* to 1.4, assuming *sbd* = 1 g cm^−3^ and *ld* = 15 cm (Eq. (1)). Several authors have used such arbitrary *sbd* and a fixed *ld* to estimate the lime requirement in tons per ha (Osmond et al., 2002, Sanchez, 2019, Yost et al., 1988). However, this practice should be avoided because it greatly affects the results. For example, a soil with *sbd* = 1.2 g cm^−3^ requires 20% more lime than one with the same chemical properties and *sbd* = 1 g cm^−3^, and the amount of lime required with *ld* = 15 cm is 25% less than with a *ld* = 20 cm.

#### 4.1.4 NuMaSS model

The Integrated Soil Nutrient Management Decision Support System (NuMaSS) was developed to provide fertilizer (N and P) and liming recommendations for acid soils with nutrient problems (Osmond et al., 2002, Walker et al., 2009). In NuMaSS, soil N, P, and acidity constraints are computed individually. Then, the final management recommendation is computed by considering the costs and benefits of different nutrient management strategies. The acidity module considers Al toxicity and deficiencies of Ca and Mg, although the main focus was on Al toxicity. Al toxicity is computed based on crop critical acidity saturation, exchangeable acidity, and *ECEC*. Default crop critical acidity saturation values for many crops and varieties were included. The lime rate was calculated with another modified Cochrane et al. (1980) formula (Eq. (4)).

$$\mathrm{LR}\left(t\;ha^{-1}\right)=lf\times \left(exch.acid-\frac{\mathrm{TAS}}{100}\times ECEC\right)+\left[10\times ECEC\times \frac{\max\left(19-TAS,0\right)}{100}\right]$$

(4) lf=2.5,ifECECclay<4.51.3,ifECECclay≥4.5

where *clay* is the clay content in the soil.

This model uses different lime factors depending on the soil’s clay activity (effective cation exchange capacity of the soil’s clay fraction). According to its authors, soils with low clay activity (i.e.*,* low *ECEC* per unit of clay) require almost twice the lime amount of soils with high clay activity (i.e., high ECEC per unit of clay) to neutralize the same amount of exchangeable acidity charges. In addition, they considered that reducing the acidity saturation below 19% requires an additional amount of lime equivalent to 10% of the *ECEC* per percentage point. The NuMaSS model predicts lime rates in tons per hectare by assuming *ld* = 15 cm and *sbd* = 1 g cm^−3^.

To test the NuMaSS model with the soil incubation studies data, the predicted *LR* was transformed from t ha^−1^ to cmol_c_ kg^−1^ (Eq. (1)). Moreover, to take advantage of all the data while being conservative in the lime requirement prediction, high clay activity (lowest *lf* and lower *LR*) was assumed when data on soil clay content were not available (Table 1, Fig. 2).

The NuMaSS formula adds much complexity to the formula of Cochrane et al. (1980). It considers that the acidity saturation response to increasing lime rates is not linear and that the response depends on a soil’s clay activity. However, in our analysis, NuMaSS consistently overpredicted the lime rates required to reach a certain level of acidity saturation (Fig. 2), particularly for low *TAS* (<10%), indicating that the second term of the formula for *TAS* < 19% should be revised or omitted. Unfortunately, the software is no longer available, and the data used to derive the formula are unavailable, so the model cannot be further scrutinized.

#### 4.1.5 Minas Gerais 5th approximation model

This Minas Gerais 5th approximation (MG5) model developed for the state of Minas Gerais, Brazil (Alvarez and Ribeiro, 1999) also has two terms, one of them deriving from the model of Cochrane et al. (1980). It considers the lime rate needed to lower the acidity saturation of the soil to a target level, as well as possible Ca and Mg deficiencies for the crop. The formula can be written as follows:

(5) $$LR\left(t\;ha^{-1}\right)=lf\times \left[exch.a{\mathrm{cid}}_{i}-\left(\frac{\mathrm{TAS}}{100}\right)\times ECEC_{i}\right]+\max\left(X-\left(exch.Ca+Mg\right),0\right)$$

$$\mathrm{lf}=0.0302+0.06532\%clay-0.000257\%clay^{2}$$

where *X* is the sum of the minimum quantity of exchangeable Ca and Mg required by the crop (estimated as 2 cmol_c_ kg^−1^ for most cereals and legumes and 3 cmol_c_ kg^−1^ for most fruits and vegetables, Alvarez and Ribeiro, 1999). Note that the second term of the formula becomes zero when the initial exchangeable Ca^2+^ and Mg^2+^ meet crop demands, while the first term is equal to the model of Cochrane et al. (1980) but with a different lime factor that depends on soil texture. The *lf* can take any value between 0 and 4, with higher values in clay soils.

The Kamprath, 1970, Cochrane et al., 1980 soil incubation studies data show very little support for such a drastic change in *lf* (Fig. 2 and Table 2). Furthermore, the addition of the second term in (Eq. (5)) has no theoretical justification because, while the carbonate of the CaCO_3_ precipitates the exchangeable aluminum (forming aluminum hydroxide), the Ca^2+^ stays in the cation exchange complex and becomes available for the crop (Sanchez, 2019). Therefore, adjusting for possible Ca and Mg deficiencies would be more appropriate when the sum of the initial exchangeable Ca^2+^ and Mg^2+^ and the Ca^2+^ or Mg^2+^ supplied by the liming material does not meet crop demand.

#### 4.1.6 LiTAS: A new model to estimate lime requirements

Defining a target acidity saturation and estimating lime rates as a function of that target is a useful concept. Presumably, new models were derived from the Cochrane et al. (1980) model because of perceived shortcomings (e.g., fixed *lf* of 1.5 or 2). However, while more complicated, the derived models did not improve the prediction accuracy (Fig. 2 and Table 2). Below we introduce LiTAS, a new lime requirement model based on *TAS* obtained from a formal mathematical derivation of the concept of acidity saturation*.* Our goal is to provide a model based on strong empirical relations that can be easily updated as more data become available.

First, let us decompose the numerator and denominator of final acidity saturation ($AS_{f}\left(\%\right)=\frac{\mathrm{exch}.acid_{f}}{\mathrm{ECE}C_{f}}\times 100\%$) into their initial values and degree of change (Eq. (6)).

(6) $$AS_{f}\left(\%\right)=\frac{\mathrm{exch}.acid_{i}-\Delta exch.acid}{\mathrm{ECE}C_{i}+\Delta ECEC}\times 100\%$$

Δ*exch. acid* is the exchangeable acidity neutralized by liming (cmol_c_ kg^−1^), and Δ*ECEC* is the change in the effective cation exchange capacity, which equals the difference between the increase in exchangeable bases (Δ*exch. bases*) minus the neutralized exchangeable acidity (Δ*exch. acid*). Δ*ECEC* is usually positive (Δ*exch. bases* > Δ*exch. acid*) because *ECEC* increases at higher pH; thus, it increases with liming (Edmeades, 1982).

(7) $$AS_{f}\left(\%\right)=\frac{\mathrm{exch}.acid_{i}-\Delta exch.acid}{\mathrm{ECE}C_{i}+\Delta exch.bases-\Delta exch.acid}\times 100\%$$

**Fig. 3:**
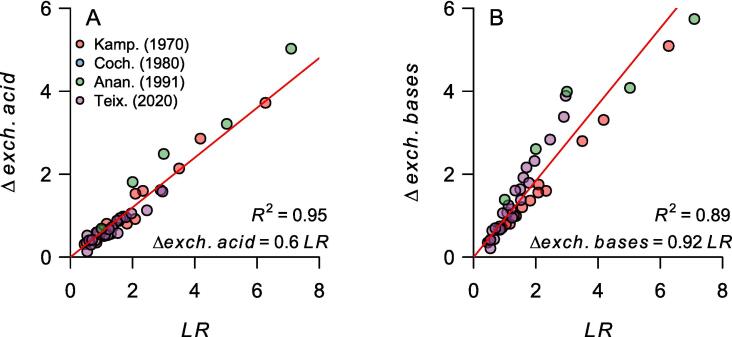
(A) Exchangeable acidity neutralized (Δ*exch. acid*, cmol_c_ kg^−1^) and (B) exchangeable bases increase (Δ*exch. bases*, cmol_c_ kg^−1^) as a function of the lime rate (*LR*, cmol_c_ kg^−1^), for soil samples with a final acidity saturation ≥ 5%. The lines are regression lines forced through the origin (equations shown in the plot). To avoid the high leverage of soil samples with the highest *LR*, *LR* and Δs were transformed with the square root before linear regression fitting, and then the coefficients estimates were back-transformed. The coefficient of determination was computed as the square of Pearson’s correlation coefficient between observed and linear regression-predicted values. Data extracted from Kamprath (1970) (Kamp.), Cochrane et al. (1980) (Coch.), Ananthacumaraswamy and Baker (1991) (Anan.), and Teixeira et al. (2020) (Teix.).

Considering that our goal is to make the final acidity saturation equal to the target acidity saturation (*AS_f_* = *TAS*), *AS_f_* can be replaced with *TAS* in Eq. (7). Then, *TAS* becomes a function of the initial soil properties (*ECEC_i_* and *exch. acid_i_*), the increase in exchangeable bases (Δ*exch. bases*), and the exchangeable acidity neutralized (Δ*exch. acid*). Therefore, to estimate the required *LR* to reach a given *TAS,* we need to find the association of Δ*exch. acid* and Δ*exch. bases* with *LR* so that the two former variables can be replaced for some function of *LR* in Eq. (7). For soils with *AS_f_* ≥ 5%, these two associations can be modeled with a linear regression without intercept (Fig. 3), despite slight but significant differences between studies. In Fig. 3B, the exchangeable bases increase per unit of applied lime is higher for Teixeira et al. (2020a) observations than for Kamprath (1970) (*P* < 0.001).

Based on this assumption, we have:

(8) Δexch.acid=a×LR

(9) Δexch.bases=b×LR

We replace the deltas in Eq. (7) with Eq. (8) and Eq. (9) to obtain:

(10) $$TAS\left(\%\right)=\frac{\mathrm{exch}.acid_{i}-a\times LR}{\mathrm{ECE}C_{i}+b\times LR-a\times LR}\times 100$$

And we solve for *LR* to get

(11) LRcmolckg-1=exch.acidi-TAS100×ECECia+TAS100×b-a

Based on the soil incubation studies data and the regression lines shown in Fig. 3, the parameter estimates for *a* and *b* were 0.60 and 0.92, respectively. These unit-less parameters were estimated using the square root of the values to reduce the leverage of very high *LR* values and then back-transformed. Note that *a*, which is the cmol_c_ of exchangeable acidity neutralized per cmol_c_ of CaCO_3_, is similar to the value reported by Yost et al. (1988), which was 0.53. These values can be updated or calibrated for a particular region. Moreover, if new evidence refutes the assumption of a linear association between *LR* and the change in exchangeable bases and acidity, all formulas from Eq. (7) onwards would need to be updated, but the framework would remain the same.

Notably, the numerator in Eq. (11) is the same subtraction term found in the model of Cochrane et al. (1980) and all other models derived from it. Hence, if Eq. (11) is rewritten by splitting the numerator and denominator, the inverse of the denominator can be interpreted as a new lime factor (*lf*), which is an inverse function of *TAS* (Eq. (12)). Although the *lf* derived in Eq. (12) is very different conceptually from the *lf* introduced by Cochrane et al. (1980; Eq. (3)), its possible values are similar to those used by previous models. Given our estimates of parameters *a* and *b*, the value of the *lf* would be between 1.5 and 1.6 for most crops.

(12) LRcmolckg-1=lf×exch.acidi-TAS100×ECECi

lf=1a+TAS100×b-alf^=10.6+TAS100×0.92-0.6

The LiTAS model has greater accuracy than the Cochrane model and all other models derived from it (Fig. 2 and Table 2). The accuracy improvement was also evident when the model was evaluated with independent data, that is when calibrating and testing the models with data from different studies (Table 2). Therefore, the LiTAS model has improved accuracy and general validity because there was no accuracy loss when fitting the model with data from one region and then making predictions for soils from another region.

### 4.2 Base saturation model

A “*base saturation*” model originally proposed by Quaggio (1983) is widely used in São Paulo state, Brazil (Sanchez, 2019, van Raij, 1996). Base saturation (*V*) is the sum of exchangeable bases over *CEC_7_*, expressed as a percentage (see section 2.1.3). The model’s formula is

(13) $$LR\left(cmol_{c}kg_{\mathrm{soil}}^{-1}\right)=CEC_{7}\times \left(V_{t}-V_{i}\right)/100$$

*V*_t_ is the target, and *V_i_* is the initial base saturation. Like *TAS*, *V_t_* is crop-specific and expresses a crop’s sensitivity to soil acidity. In São Paulo, Brazil, *V_t_* is 50% for most cereals and legumes, including maize, wheat, rice, sorghum, soybeans, and beans, while it is between 60% and 80% for most fruits and vegetables (Alvarez and Ribeiro, 1999, Sanchez, 2019).

Since *CEC_7_* is, in principle, not affected by liming (contrary to *ECEC*), *CEC_7_* can be distributed to *V_t_* and *V_i_* in Eq. (13) and canceled out. Thus, the lime requirement estimated by this model is equal to the difference between the target and the initial sum of exchangeable bases:

(14) $$LR\left(cmol_{c}kg_{\mathrm{soil}}^{-1}\right)=exch.bases_{t}-exch.bases_{i}=\Delta exch.bases$$

**Fig. 4:**
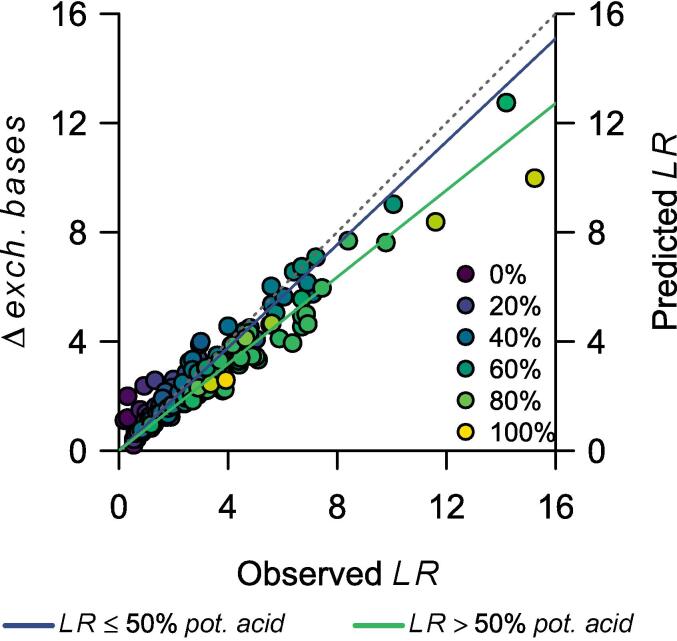
Difference in exchangeable bases before and after liming (Δ*exch. bases* = *exch. bases*_*i*_ - *exch. bases*_*f*_, cmol_c_ kg^−1^) as a function of the observed lime rate (*LR*, cmol_c_ kg^−1^). Δ*exch. bases* equals the predicted lime rate by the base saturation model. The color of the points represents the ratio between *LR* (cmol_c_ kg^−1^) and the potential acidity of the soil (pot. acid = *CEC*_7_ – *exch. bases*_*i*_). The gray dashed line is the identity function (Δ*exch. bases* = *LR*). The solid lines are regression lines forced through the origin. The blue line is for soil samples with *LR* ≤ 50% pot. acid (Δ*exch. bases* = *LR* × 0.95(±0.05)). The green line is for *LR* > 50% pot. acid (Δ*exch. bases* = *LR* × 0.8(±0.03)). The data was extracted from Kamprath (1970), Teixeira et al. (2020), and Ananthacumaraswamy and Baker (1991). Soil samples with lime rates higher than the potential acidity were omitted.

The base saturation model implicitly assumes that all Ca^2+^ (and Mg^2+^) positive charges from the lime become part of the exchangeable complex (Quaggio, 1983). Fig. 3B shows the association between observed *LR* and Δ*exch. bases* for soil samples with *AS*_f_ ≥ 5%. Fig. 4 expands that association to all soil samples with *LR* equal to or lower than the initial potential acidity (*pot. acid_i_* = *CEC_7_* – *exch. bases_i_*). It excludes soil samples with *LR* > *pot. acid_i_* because the increase in exchangeable bases cannot be greater than what the cation exchange complex can take. When *LR* ≤ 50% *pot. acid_i_*, there is almost a one-to-one association between the lime rate and the increase in exchangeable bases charges (Δ*exch. bases* = *LR* × 0.95(±0.05) ∀ *LR* < 0.5 × *pot. acid_i_*, Fig. 4), supporting the base saturation model assumption. However, as the lime rate approaches the potential acidity, that association becomes weaker (Δ*exch. bases* = *LR* × 0.8(±0.03) ∀ 0.5 × *pot acid_i_* < *LR* < *pot acid_i_*, Fig. 4). Thus, this model yields a final base saturation close to the target when *V_t_* ≤ 50%, but it does not perform well at higher base saturation targets (Table 2). Consequently, in the future, a liming correction factor (*lf*) that depends on *V*_t_ could be considered for the model. For example, the *lf* could be 1.05 when *V*_t_ ≤ 50% (i.e., 1/0.95) and then slightly increase as *V_t_* approaches 100%, with a maximum *V*_t_ of 1.25 (i.e., 1/0.8).

### 4.3 Target pH model

#### 4.3.1 Teixeira model

Teixeira et al. (2020b) developed a lime requirement model that targets raising the soil pH to a level considered optimal for crop production. The model is based on four nonlinear models that relate the difference between the initial pH and two target pHs (5.8 and 6) with either organic matter content (*OM,* g kg^−1^) or potential acidity (Eq. (15)). It also considers that the lime rate must be greater than the Ca and Mg requirement of the crop (*X*) and lower than the potential acidity of the soil (*pot. acid_i_*). Thus, the estimated lime requirement results from a series of rules such that it selects the lowest *LR* from the four nonlinear models that is higher than *X* and lower than *pot. acid_i_*. When no model returns a lime rate higher than *X*, the estimated *LR* is *X*. If the selected *LR* (either from the models or *X*) is greater than the initial potential acidity of the soil, the estimated *LR* equals *pot. acid_i_*. This model always recommends liming because the Ca and Mg available in the soil are ignored when computing crop requirements, and it thus assumes that all Ca and Mg must be provided by liming. Therefore, the minimum lime rate is *X* (Ca and Mg crop requirements), except when *X* is higher than *pot. acid*, in which case *LR = pot. acid.*

$$LR_{5.8OM}=0.0699\times {\left[\left(5.8-pH\right)OM\right]}^{0.9255}$$

$$LR_{5.8PA}=0.375\times {\left[\left(5.8-pH\right)pot.acid\right]}^{0.9127}$$

$$LR_{6OM}=0.1059\times {\left[\left(6-pH\right)OM\right]}^{0.8729}$$

(15) $$LR_{6PA}=0.4558\times {\left[\left(6-pH\right)pot.acid\right]}^{0.9162}$$

**Fig. 5:**
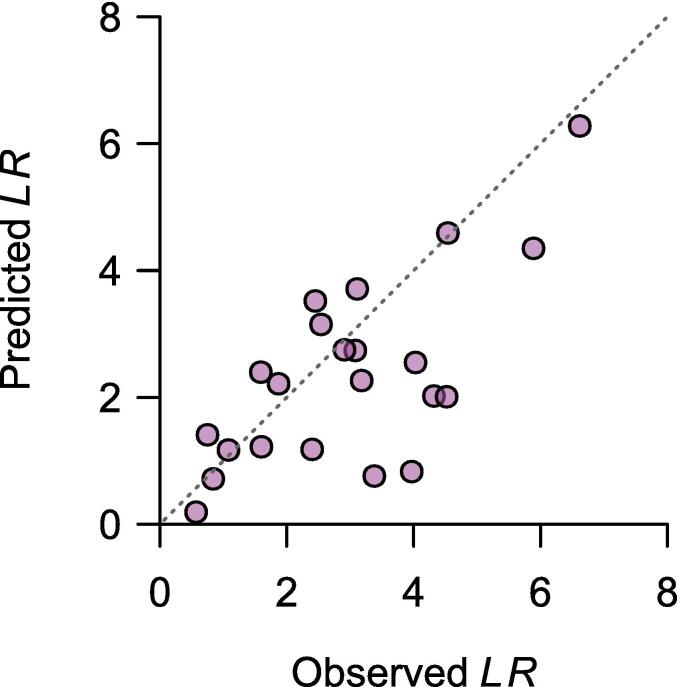
Predicted lime rate (*LR*) to reach a pH of 5.8 by Teixeira et al. (2020b) as a function of the observed *LR* that resulted in such a pH. The gray dashed line is the identity function (Predicted *LR* = Observed *LR*).

The model parameters were calibrated with the same soil incubation study data from Teixeira et al. (2020a). However, these authors excluded data from five soils from the calibration because they considered that these observations deviated too much from the nonlinear regression models compared to the data from other soils. We tested the model with six-fold cross-validation using data from Teixeira et al. (2020a), including the observations excluded in the original calibration by Teixeira et al. (2020b; Fig. 5). The target pH model has much lower accuracy than all other models above (Table 2). Furthermore, as the model selects the minimum *LR* from the nonlinear models instead of the average, it often underpredicts *LR*.

The Teixeira et al. (2020b) model is the most recent of a large number of regression models based on a target pH developed for acid soils in Brazil (see, for example, Combatt Caballero et al., 2019). These models use linear or nonlinear regression with variables such as ΔpH, organic matter, potential acidity, and base saturation to predict lime rates for a particular region. However, when tested with an independent dataset, these models have low accuracy (Teixeira et al., 2020a), which might be related to the many factors affecting soil pH. Most likely, no simple model can predict soil pH responses to liming for different soil types with regular soil testing data. Incorporating additional soil properties that measure the soil acid-base buffering capacity into routine soil tests could help develop better predictive liming-soil pH models (Yang et al., 2020).

#### 4.3.2 Buffer capacity models

One of the most straightforward and oldest methods to determine the lime required to raise the pH to a specific level is to take a soil sample and measure the quantity of base required to produce that pH change by a slow titration in the lab (Pierre and Worley, 1928). The amount of lime required to be added to the soil per unit of pH increase is known as the lime-based buffer capacity of the soil, which is calculated from the slow-titration curve, adjusting for soil bulk density and lime incorporation depth (Strawn et al., 2019). However, this method is time-consuming and requires great experimental precision, which makes it impractical for soil-testing purposes across large areas (Strawn et al., 2019). Therefore, the calibration curves from the titration of a few soils are commonly used to estimate the lime requirements of similar soils from the same geographic region, and buffer capacity information is not available in spatial databases of soil properties that cover the tropics (Hengl et al., 2017, Leenaars et al., 2014, Miller et al., 2021). As we only focused on lime requirement models based on soil properties for which estimates are available in spatial databases for any location, we did not assess the accuracy of the buffer capacity method, but we still mentioned it because of its widespread use and relevance (Jansen van Rensburg et al., 2009, Nelson et al., 2010, Taye et al., 2020).

## 5 Case study

We computed lime requirements for 303 African soils of pH between 3.5 and 6.5 (Supplementary Fig. 2 and Supplementary Table 1) with the models reviewed in Section 4 for two representative crops with different acidity tolerance. We selected maize as the more tolerant crop and groundnut as the more sensitive crop and defined the target soil properties based on the values suggested by Alvarez and Ribeiro (1999). A 15% target acidity saturation (*TAS*) and a 50% target base saturation (*V*_t_) were defined for maize, and a 5% *TAS* and 70% *V*_t_ for groundnut. Although we refer to two specific crops, these are common critical acidity values of many other cereal and legume crops. Models targeting pH were not included because these models are location-specific and have low accuracy when extrapolated to other regions (Fig. 5 and Table 2). Lime rates were computed in cmol_c_ kg^−1^ because only 27% of these soil profiles had soil bulk density data. We used these lime rate estimates to illustrate the magnitude of variation in lime requirements between the different models. We first compared models with the same target soil chemical property (i.e., acidity saturation models) and second two models with different targets (i.e., one acidity saturation vs. one base saturation model).

Of the acidity saturation models, Kamprath, Cochrane, ACID4, and LiTAS estimated very similar lime rates, while the NuMaSS and Minas Gerais 5th approximation predicted larger lime rates (Supplementary Figs. 3 and 4). Despite the high correlation and similarities between the first group of models mentioned above, there were some consistent differences. For instance, the Kamprath model estimated higher lime rates than the other three models in all soil samples for the sensitive crop and in most soils for the tolerant crop, with differences up to 3.9 cmol_c_ kg^−1^. In contrast, the LiTAS model estimated lower lime rates than other models on average and for most soils, with larger differences for the more sensitive crop (Supplementary Figs. 3 and 4).

**Fig. 6:**
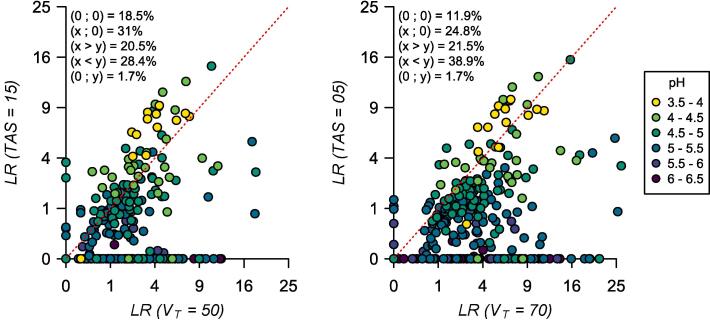
Estimated lime rates (*LR*, cmol_c_ kg^−1^) for 303 African soils with pH between 3.5 and 6.5, two target soil chemical properties: a target base saturation (*V*_t_, x-axis) and a target acidity saturation (*TAS*, y-axis), and two representative crops: (A) maize (*TAS* = 15% and *V*_t_ = 50%) and (B) groundnut (*TAS* = 5% and *V*_t_ = 70%). The red dashed line is the identity function (*LR*_(*TAS*)_ = *LR*_(*V*t)_). The values inside the plot indicate the fraction of soils in a specific scatter plot position: the origin (0;0), the x-axis (x;0), between the x-axis and the identity function (x > y, lower triangle), between the identity function and the y-axis (x < y, upper triangle), and the y-axis (0;y). Lime rates based on *TAS* were predicted with the acidity saturation model presented in Eq. (12), and *LR* based on *V*_t_ with Quaggio (1983).

We also contrasted the lime rates estimated by the LiTAS model and Quaggio’s (1983) base saturation model for the two crop examples and the 303 African soils. The most striking difference was that the base saturation model recommended liming for many soils that did not require liming according to the acidity saturation model (Fig. 6). For instance, both models agreed that no lime was needed for maize in 18.5% of the soils. However, 31% of the soils required liming according to the base saturation model but did not need to be limed according to the acidity saturation model. In contrast, only 1.7% of the soils required liming based on acidity saturation but did not require lime based on their base saturation (Fig. 6A). The disagreement on the soils requiring liming based on the different target soil properties resulted from the large proportion of soils with an acidity saturation lower than 15% but a base saturation lower than 50% (Supplementary Fig. 5).

Moreover, the base saturation model predicted lime rates as high as 12 cmol_c_ kg^−1^ for soils with a pH higher than 6, indicating that even these soils can have a low base saturation, while virtually no soil with such a pH required liming based on acidity saturation (Supplementary Fig. 6). For soils that required liming based on both their acidity and base saturation, the estimated lime rates by the two models were weakly correlated (*r* = 0.43) but comparable in magnitude (mean difference = 0.47 cmol_c_ kg^−1^). The acidity saturation model predicted higher lime rates in soils with very low pH for the more tolerant crop (Fig. 6A), but the rates were more similar for the more sensitive crop (Fig. 6B). Conversely, the base saturation model predicted higher lime rates for most soils with a pH above 5, particularly for the more sensitive crop (Fig. 6B).

## 6 Discussion

### 6.1 Model comparison

We showed important differences between models in the accuracy of the required lime rates. When the target is to reduce the Al toxicity by neutralizing its acidity saturation to a certain level, both Kamprath, 1970, Cochrane et al., 1980 models were reasonably accurate. Nevertheless, our new model (LiTAS) offers improved accuracy and the advantage of being based on a formal mathematical derivation that can be expanded. Similarly, the base saturation model also had high prediction accuracy, particularly for target base saturation levels of around 50%. In contrast, models based on a target pH can only deliver accurate results when coupled with additional soil tests, such as slow titrations that estimate the soil buffer capacity. These tests need to be developed locally, and the calibrated model is only useful for similar soils of the same geographic region (Sims, 1996; Strawn et al., 2019).

LiTAS was the only model based on a target acidity saturation (*TAS*) with greater accuracy than the original Cochrane et al. (1980) model. The authors of the ACID4, NuMaSS, and MG5 models claimed that they modified the Cochrane et al. (1980) model to improve the accuracy for their target region. Unfortunately, we did not have access to the data used by these authors, but we suspect that these more complex models suffer from overfitting the datasets used to develop them. In other words, they may have performed better for particular datasets from particular regions, but this has come at the expense of general validity. Conversely, the LiTAS model was robust to changes in the data used to calibrate it, being more accurate than previous models even when evaluated with an independent dataset, that is, data from a study that was not used to calibrate it. The general validity we observed in the new and other acidity saturation models might be related to the strong association between lime rates and the increase or decrease in exchangeable acidity and bases, which were consistent for all soils from the different regions included in the analysis.

We observed a small incubation study effect in our model accuracy assessment. This might be a consequence of the soil region (parental material) or, more likely, because of the incubation study per se (differences in the liming material or soil incubation method). Experimental results have an error component, including systematic errors that are consistent within one experiment but differ between experiments, introducing statistical bias. This bias can be reduced with standardized procedures. However, lime incubation studies are not fully standardized and differ in the incubation time and temperature, liming materials, and water additions, among other variables (Salinas, 1978). For instance, we excluded data from an incubation study in which control treatments had significantly more exchangeable Ca^2+^ and less exchangeable acidity than the initial conditions, likely a consequence of using tap water rather than distilled water to keep the soil samples moist during the incubation (Deressa et al., 2020). A more thorough standardization of experimental procedures for measuring liming effects would help the development of general models for lime requirement estimation.

A novel feature of the LiTAS model is that the lime factor (*lf*) is a continuous function of *TAS*. The Cochrane et al. (1980) model modifies the *lf* depending on *TAS* and the initial acidity saturation, using a discontinuous rule with two fixed levels of *lf*. However, the proposed rule did not always improve accuracy, not even for Cochrane et al.’s own data. In the MG5 and NuMaSS methods, the *lf* depends on clay content or activity. Our review does not show evidence for a need to adjust the *lf* as a function of clay, despite the wide range of clay content and soils included in the four soil incubation studies used here. Adjusting the *lf* and lime rates by clay content might be a workaround to account for differences in soil bulk density when the method returns lime rates in tons per ha without directly including the soil bulk density in the formulas. Nevertheless, clay type and content could be considered in future corrections of the *TAS* method, particularly if there are high deviations in the association between lime rate and Δ*exch. acid* and Δ*exch. bases*.

It seems counterintuitive that while both the acidity saturation and base saturation models were highly accurate for their target, the lime requirements they predicted were sharply different. These differences highlight the importance of identifying the soil chemical property most associated with the crop yield response to liming. Tropical soils can have several acidity problems affecting crop growth (Kamprath, 1984, Sanchez, 2019). It might be that reaching a given level for some property, such as a base saturation of 50% or a pH of 5.5, guarantees that all soil acidity problems are solved without leading to overliming problems. However, this approach can also result in lime requirement estimates that are much too high (Farina and Channon, 1991, Smyth and Cravo, 1992), which might be particularly problematic when lime is expensive and its manipulation cumbersome. The alternative is to target the most limiting factor for crop yield, which is frequently Al toxicity in acid tropical soils (Sanchez, 2019). However, this approach can underpredict lime requirements when Al toxicity is the only target but not the acidity problem most limiting crop yields. A comprehensive approach would predict the lime rate needed to tackle every acidity problem while considering other management alternatives. However, crop responses to other acidity problems, such as Ca and Mg deficiencies, are unclear, and their liming requirements have not been defined. Thus, more research on crop responses to lime in soils with these specific acidity problems is needed to develop a lime requirement method that tackles them all.

### 6.2 Model applications

Lime requirement models can be useful for strategic research on potential lime use in tropical regions where liming is still rare and experimental evidence is scarce (Crawford et al., 2008). These models estimate the lime rate needed to reach a target soil condition based on readily available standard soil data (Hengl et al., 2017, Miller et al., 2021). Such information could be used with the crop response to that soil condition to estimate the effect of liming on crop yield. For instance, there is ample evidence of the association between acidity saturation and crop yields (Abruña et al., 1969, Farina and Channon, 1991, Lollato et al., 2019, Smyth and Cravo, 1992). Therefore, the expected yield response to lime can be predicted by estimating what fraction of the maximum yield is observed at the current acidity saturation level while assuming that the final yield after liming is the inverse of that fraction. If data on lime and grain prices are available, such functions can be used to get a first approximation of the profitability of liming (Bonilla-Cedrez et al., 2021). Such analysis can help identify regions where liming investments might be more successful, pinpointing national governments and private sector efforts.

However, this does not mean that the quality of the readily available soil data used by the models reviewed here is sufficient for farm-level recommendations (Vanlauwe et al., 2019). Soil pH is the most commonly measured soil property related to soil acidity, and it is, therefore, likely that estimates of pH in spatial databases of soil properties are relatively accurate. However, soil pH alone cannot be used to estimate lime requirements (Sanchez, 2019, Sims, 1996). Soil pH can be used only to detect potential soil acidity problems because not all tropical soils with low pH (pH < 5.5) might require lime. Measuring and mapping other key soil chemical properties, such as exchangeable acidity and *ECEC*, could help the development of site-specific lime recommendations. In the meantime, farm-level lime requirement estimates need to be informed by locally measured soil properties and could also consider additional local soil-quality indicators, such as soil color, soil texture, or the presence of specific plant species (Mairura et al., 2007). The soil properties used by the lime requirement models reviewed here are wet-lab measurements, which are costly and may be inaccessible for farmers in the tropics. Therefore, farmers in the tropics could benefit from cost-effective, quick tests for lime requirement prediction, but these need to be developed locally.

## 7 Conclusions

Liming can increase crop productivity in acid soils, but the lime rate required to achieve this is unknown for many tropical regions. While lime requirement models could be very useful, the proliferation of models introduces uncertainty about which model to use. We discussed the strengths and weaknesses of various lime requirement models that can be used with data readily available in spatial soil databases. We showed important differences in the amount of lime required according to these models, especially when considering different target soil chemical properties. LiTAS, the new acidity saturation model introduced here, is more accurate than all prior models across many acid tropical soils from different regions and can effectively estimate the lime rate required to lower the acidity saturation to a specific target. This model could be incorporated into more comprehensive models once lime rates needed for other acidity problems are well established.

## Declaration of Competing Interest

The authors declare that they have no known competing financial interests or personal relationships that could have appeared to influence the work reported in this paper.

## Data Availability

All the data and code used in the paper is available on GitHub (https://github.com/cropmodels/limer).
