# Supplementary material for: Estimating lime requirements for tropical soils: Model comparison and development
Source: Geoderma. 2023 Apr;432:116421. doi: 10.1016/j.geoderma.2023.116421 (PMC10033874; doi:10.1016/j.geoderma.2023.116421)
Supplement: Supplementary data 1 [file mmc1.docx]

**SUPPLEMENTARY FIGURES**

**Estimating lime requirements for tropical soils: Model comparison and development**

Fernando Aramburu Merlos^* a,b^, João Vasco Silva ^c,^, Frédéric Baudron ^c^, and Robert J. Hijmans^a^

^a^ Department of Environmental Science and Policy, University of California Davis, Davis, California, United States of America.

^b^ Instituto Nacional de Tecnología Agropecuaria (INTA), Unidad Integrada Balcarce, Balcarce, Buenos Aires, Argentina.

^c^ International Maize and Wheat Improvement Center (CIMMYT), Harare, Zimbabwe

^*^Corresponding author. Email: faramburumerlos@ucdavis.edu


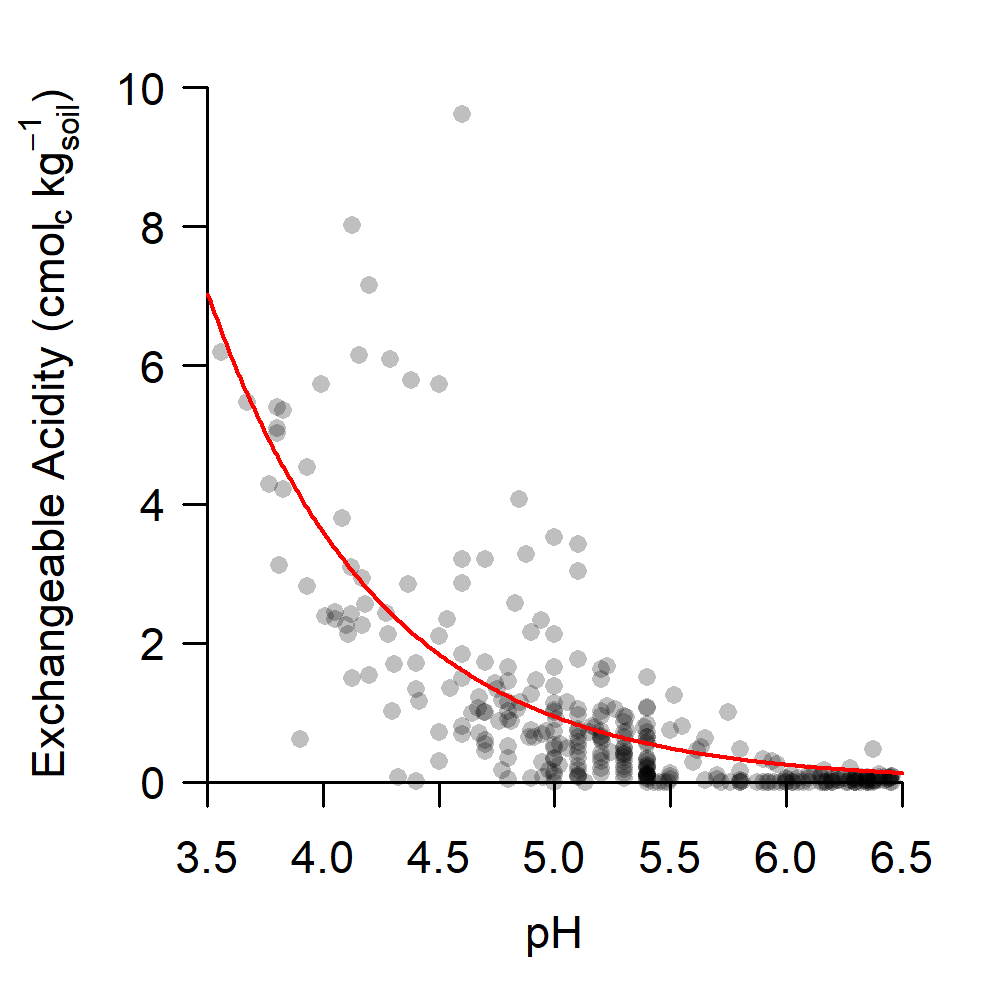


Supplementary Figure 1. Soil pH measured in water as a function of the exchangeable acidity extracted with 1 M KCl unbuffered salt solution. The data are for a depth of 0–20 cm in 303 soil samples across Africa (Leenaars et al., 2014) (see Supplementary Figure 2 and Supplementary Table 1). The red line is a negative exponential regression line $Exchangeable acidity \left( cmol_{c}kg_{soil}^{-1} \right)=765 e^{-1.34 pH}$


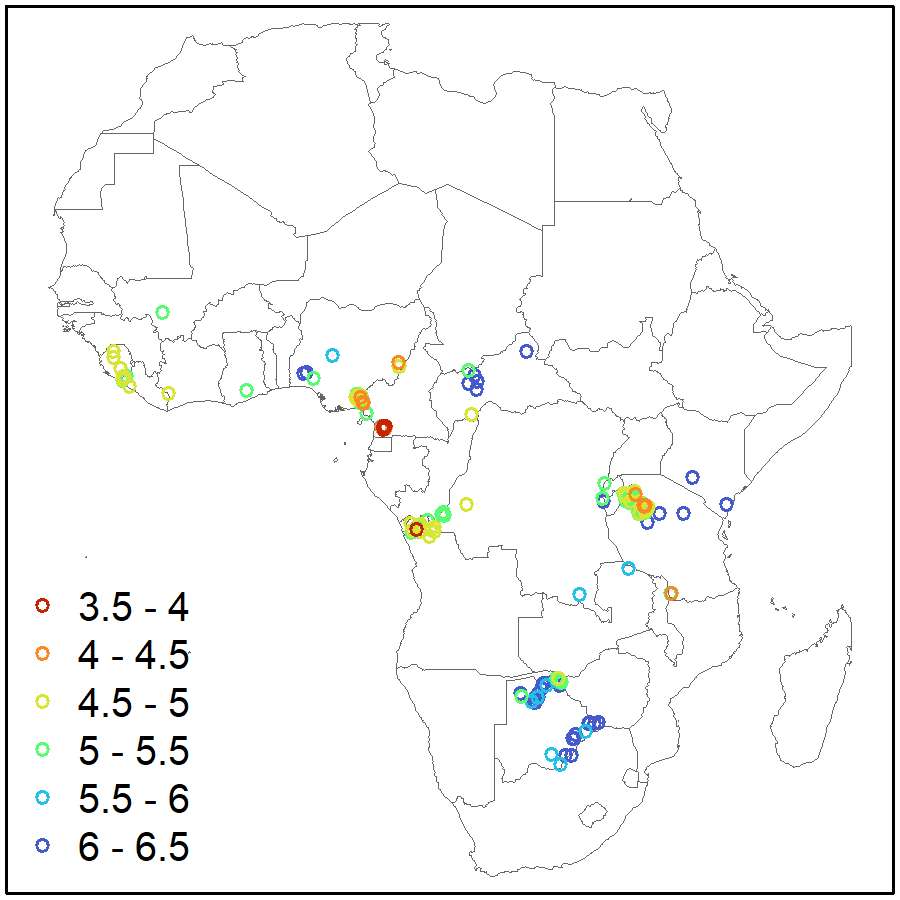


Supplementary Figure 2. Location of the 303 soil samples used for the case study. Soil data were extracted from the Africa Soil Profile Database (Leenaars et al., 2014). The color of the points indicates the pH (H_2_O) of the soil measured in water for a 0 – 20 cm depth.

Supplementary Table 1. Soil properties summary of the 303 soil samples used for the case study. Soil data were extracted from the Africa Soil Profile Database (Leenaars et al., 2014).

|  |  | Quantile | | | | |
| --- | --- | --- | --- | --- | --- | --- |
| Soil property | **Unit** | **Min** | **25%** | **50%** | **75%** | **Max** |
| pH (H_2_O) |  | 3.56 | 4.8 | 5.2 | 5.89 | 6.46 |
| Acidity saturation | % | 0 | 1.75 | 15.33 | 50.2 | 94.77 |
| Base saturation | % | 0.57 | 16.85 | 28.88 | 45.33 | 99.89 |
| Exchangeable acidity (Al^3+^ + H^+^) | cmol_c_ kg^-1^ | 0 | 0.05 | 0.46 | 1.14 | 9.62 |
| Exchangeable Ca^2+^ | cmol_c_ kg^-1^ | 0.04 | 0.72 | 1.5 | 3.48 | 31.65 |
| Exchangeable Mg^2+^ | cmol_c_ kg^-1^ | 0 | 0.3 | 0.53 | 1.36 | 20.6 |
| Effective cation exchange capacity | cmol_c_ kg^-1^ | 0.28 | 1.92 | 3.07 | 5.74 | 47.15 |
| Cation exchange capacity | cmol_c_ kg^-1^ | 1 | 4.69 | 8 | 13.32 | 76.6 |
| Clay content | % | 0 | 10.75 | 22 | 35.5 | 82.2 |
| Organic matter | % | 0 | 0.93 | 1.72 | 3.45 | 2.51 |


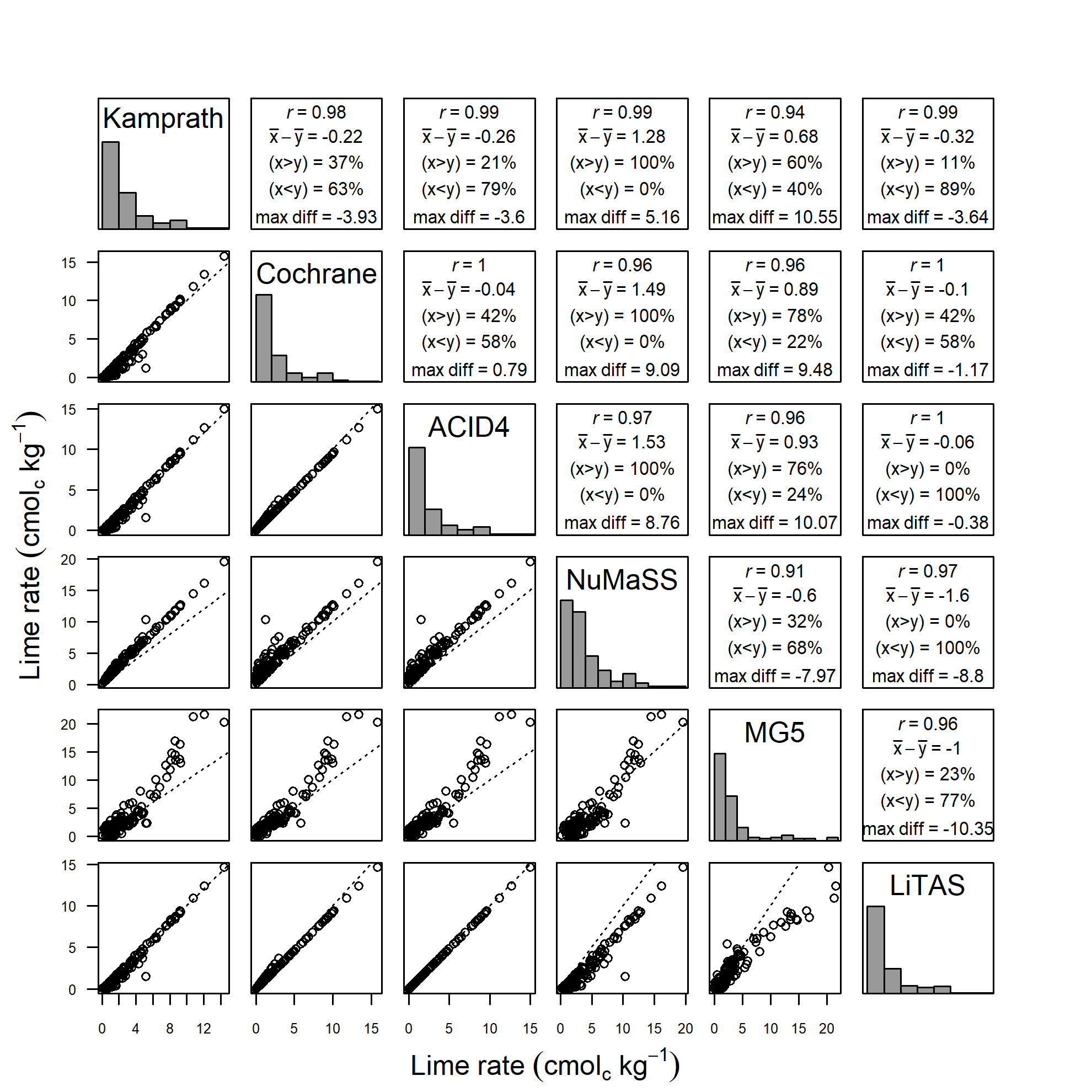


Supplementary Figure 3. Estimated lime rates (cmol_c_ kg^-1^) for 153 African soils with an Acidity Saturation greater than 15%, as predicted by different acidity saturation models and a common target acidity saturation of 15%. The model names are indicated in the diagonal of the plot matrix, together with the frequency distribution of its lime rate estimates. A model’s lime rate estimates become the x values of the plots in the same column and the y values of the plots in the same rows. For example, the scatter plot of the first column, second row, shows the lime rates estimated by the Kamprath method on the x-axis and the ones by the Cochrane method on the y-axis. The dashed lines in the scatter plots of the lower panels are the identity function (x = y). The upper diagonal shows the Pearson correlation coefficients (r), the average difference between lime rates (x̅ -y̅), the percentage of times a model predicted greater or lower rates and the maximum difference between model estimates.


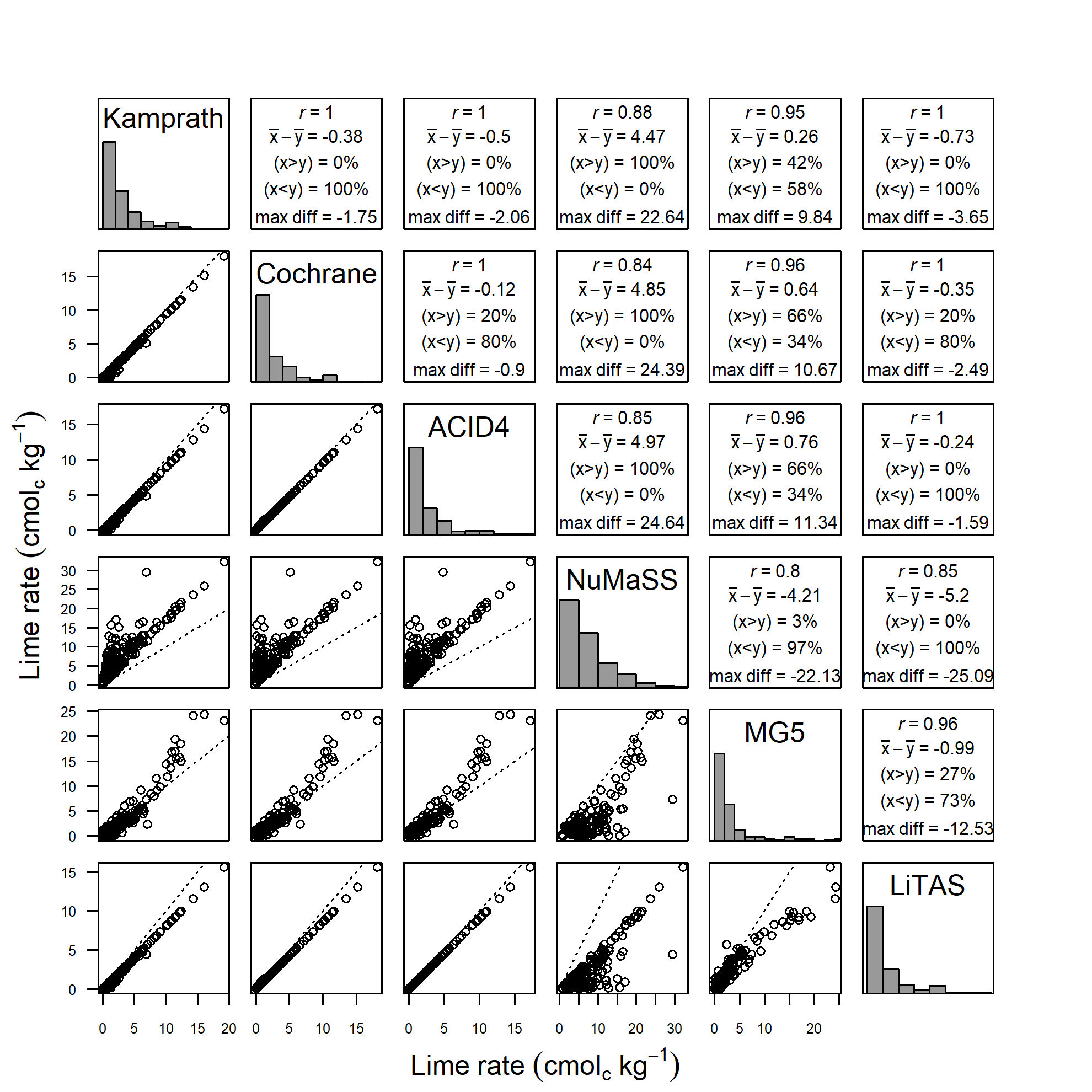


Supplementary Figure 4. Estimated lime rates (cmol_c_ kg^-1^) for 192 African soils with an Acidity Saturation greater than 5%, as predicted by different acidity saturation models and a common target acidity saturation of 5%. The model names are indicated in the diagonal of the plot matrix, together with the frequency distribution of its lime rate estimates. A model’s lime rate estimates become the x values of the plots in the same column and the y values of the plots in the same rows. For example, the scatter plot of the first column, second row, shows the lime rates estimated by the Kamprath method on the x-axis and the ones by the Cochrane method on the y-axis. The dashed lines in the scatter plots of the lower panels are the identity function (x = y). The upper diagonal shows the Pearson correlation coefficients (r), the average difference between lime rates (x̅ -y̅), the percentage of times a model predicted greater or lower rates and the maximum difference between model estimates.


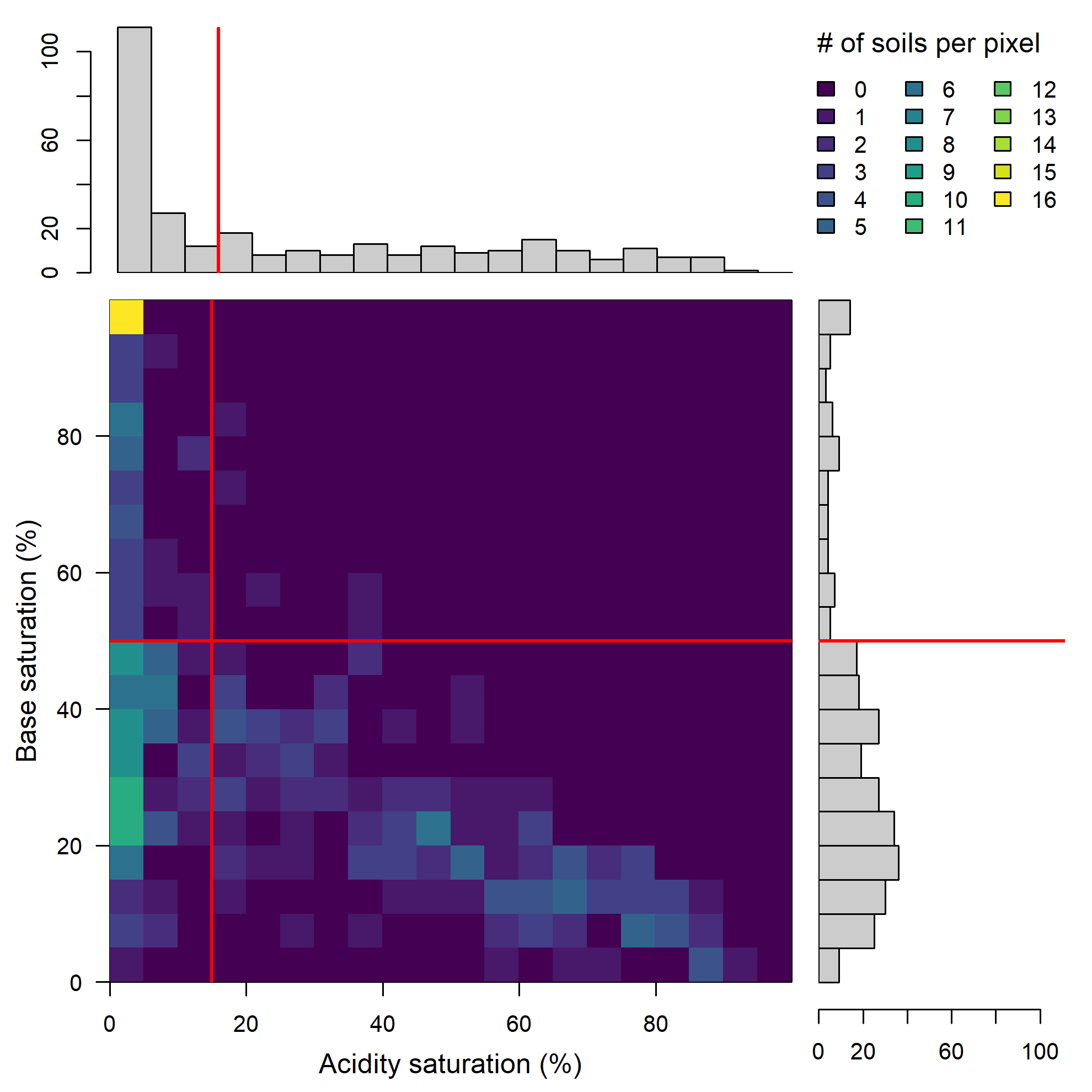


Supplementary Figure 5. Frequency distribution of the acidity saturation (%) and base saturation (%) values of the 303 soil samples used for the case study. In the 2D histogram plot, the lighter the color, the higher number of soils. The red lines show the cut-off points below or above which liming is required for maize (*i.e.*, 15% acidity saturation and 50% base saturation). Therefore, for example, liming is recommended according to both soil properties targets for the soils located in the lower-right corner. Soil data were extracted from the Africa Soil Profile Database (Leenaars et al., 2014).


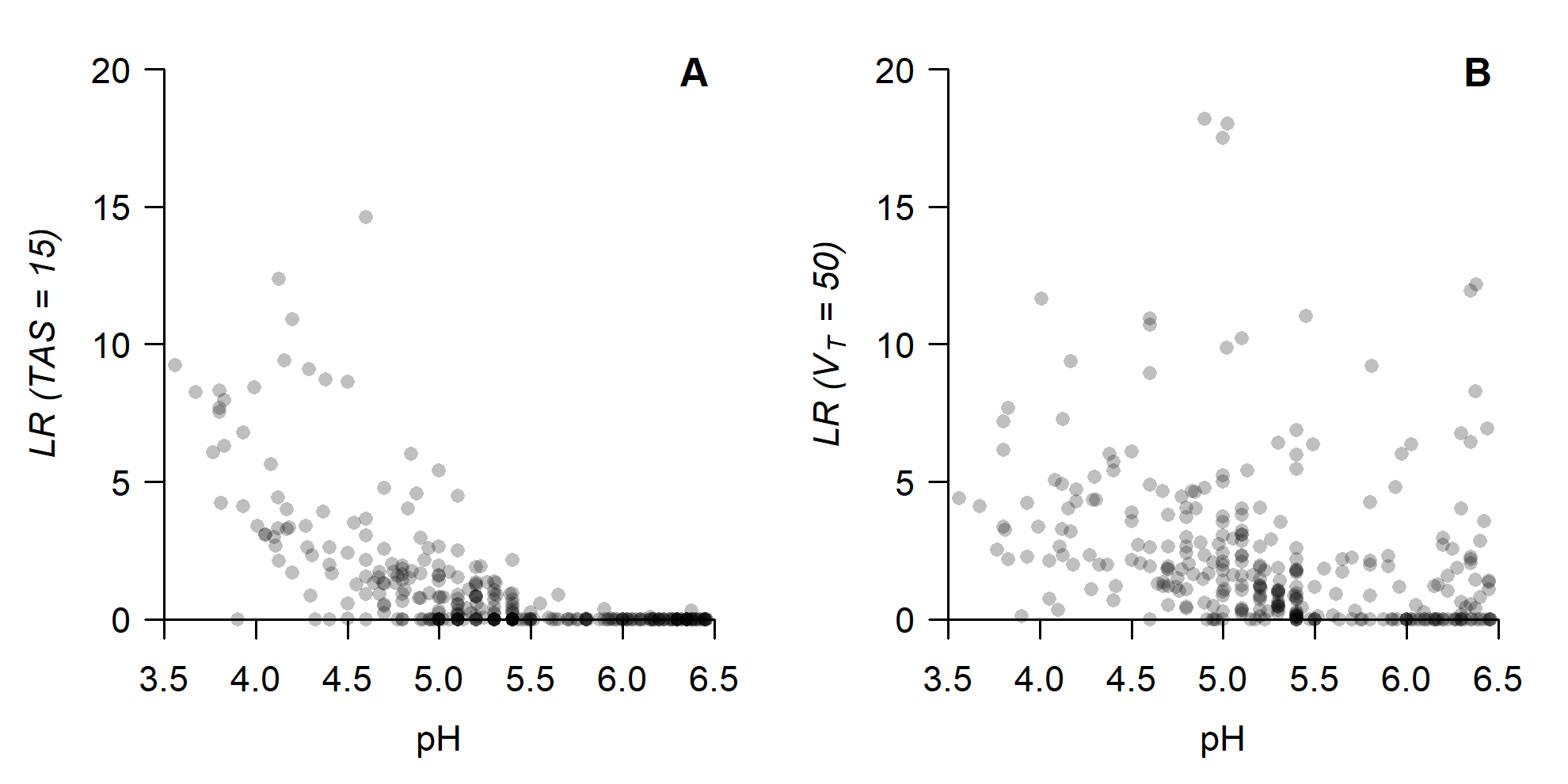


Supplementary Figure 6. Predicted lime rates (*LR*, cmol_c_ kg^-1^) as a function of soil pH (measured in water) for 303 African soils with pH between 3.5 and 6.5 and two target soil chemical properties: a target acidity saturation (*TAS*) of 15% and a target base saturation (*V*_t_) of 50%. Lime rates for *TAS*  = 15% were predicted with the acidity saturation model presented in Eq. 12, and *LR* for *V*_t_ = 50% with Quaggio (1983).
